# On a conjecture of R. Brück and some linear differential equations

**DOI:** 10.1186/s40064-015-1530-5

**Published:** 2015-12-01

**Authors:** Hong Yan Xu, Lian Zhong Yang

**Affiliations:** Department of Informatics and Engineering, Jingdezhen Ceramic Institute, Jingdezhen, 333403 Jiangxi China; Department of Mathematics, Shandong University, Jinan, 250100 Shandong People’s Republic of China

**Keywords:** Entire function, Brück conjecture, Difference polynomial, 39B32, 30D 35

## Abstract

In this paper, we mainly investigate the Brück conjecture concerning entire function *f* and its differential polynomial $L_1(f)=a_k(z)f^{(k)}+\cdots +a_0(z)f$ sharing an entire function $\alpha (z)$ with $\sigma (\alpha )\le \sigma (f)$, by using the theory of complex differential equation.

## Introduction and some results

It is assumed that the reader is familiar with the standard symbols and fundamental results of Nevanlinna value distribution theory of meromorphic functions. For a meromorphic function *f* in the whole complex plane $\mathbb {C}$, we shall use the following standard notations of the value distribution theory:

$$\begin{aligned} T(r,f),m(r,f), N(r,f),\overline{N}(r,f),\ldots \end{aligned}$$

(see Hayman 1964; Yang 1993; Yi and Yang 2003, 1995). We use *S*(*r*, *f*) to denote any quantity satisfying $S(r,f)=o(T(r,f)),$ as $r\rightarrow +\infty$, possibly outside of a set with finite measure. A meromorphic function *a*(*z*) is called a small function with respect to *f* if $T(r,a)=S(r,f)$. In addition, we will use the notation $\sigma (f), \mu (f)$ to denote the order and the lower order of meromorphic function *f*(*z*), which are defined by

$$\begin{aligned} \sigma (f)=\limsup _{r\rightarrow +\infty }\frac{\log T(r,f)}{\log r} =\limsup _{r\rightarrow +\infty }\frac{\log \log M(r,f)}{\log r}, \end{aligned}$$

and

$$\begin{aligned} \mu (f)=\liminf _{r\rightarrow +\infty }\frac{\log T(r,f)}{\log r} =\liminf _{r\rightarrow +\infty }\frac{\log \log M(r,f)}{\log r}, \end{aligned}$$

where $M(r,f)=\max _{|z|=r}|f(z)|$. We also use $\tau (f)$ to denote the type of an entire function *f*(*z*) with $0<\sigma (f)=\sigma <+\infty$, which is defined to be (see Hayman 1964)

$$\begin{aligned} \tau (f)=\limsup _{r\rightarrow +\infty }\frac{\log M(r,f)}{r^\sigma }. \end{aligned}$$

We use $\sigma _2(f)$ to denote the hyper-order of *f*(*z*), which is defined to be (see Yi and Yang 2003, 1995)

$$\begin{aligned} \sigma _2(f)=\limsup _{r\rightarrow +\infty }\frac{\log \log T(r,f)}{\log r}. \end{aligned}$$

Let *f*(*z*) and *g*(*z*) be two nonconstant meromorphic functions, for some $a\in \mathbb {C}\cup \{\infty \}$, if the zeros of $f(z)-a$ and $g(z)-a$ (if $a=\infty$, zeros of $f(z)-a$ and $g(z)-a$ are the poles of *f*(*z*) and *g*(*z*) respectively) coincide in locations and multiplicities we say that *f*(*z*) and *g*(*z*) share the value *aCM* (counting multiplicities) and if coincide in locations only we say that *f*(*z*) and *g*(*z*) share *aIM* (ignoring multiplicities).

Rubel and Yang (1977) proved the following result.

### **Theorem 1.1**

 Rubel and Yang (1977). *Let f be a nonconstant entire function. If fand *$f'$* share two finite distinct values CM, then*$f\equiv f'$.

In 1996, Brück proposed the following conjecture Brück (1996):

### **Conjecture 1.1**

 Brück (1996).* Let f be a non-constant entire function. Suppose that *$\sigma _2(f)$*is not a positive integer or infinite, if f and*$f'$*share one finite value aCM, then*

$$\begin{aligned} \frac{f'-a}{f-a}=c\end{aligned}$$

*for some non-zero constant c*.

Gundersen and Yang (1998) proved that Brück conjecture holds for entire functions of finite order and obtained the following result.

### **Theorem 1.2**

[Gundersen and Yang (1998), Theorem 1]. * Let f be a nonconstant entire function of finite order. If f and *$f'$*share one finite value aCM, then *$\frac{f'-a}{f-a}=c$*for some non-zero constant c*.

The shared value problems related to a meromorphic function *f* and its derivative $f^{(k)}$ have been a more widely studied subtopic of the uniqueness theory of entire and meromorphic functions in the field of complex analysis (see Chen et al. 2014; Li and Yi 2007; Liao 2015; Mues and Steinmetz 1986; Zhang and Yang 2009; Zhang 2005; Zhao 2012).

Li and Cao (2008) improved the Brück conjecture for entire function and its derivation sharing polynomials and obtained the following result:

### **Theorem 1.3**

 Li and Cao (2008). *Let *$Q_1$*and*$Q_2$*be two nonzero polynomials, and let P be a polynomial. If f is a nonconstant entire solution of the equation*

$$\begin{aligned} f^{(k)}-Q_1=(f-Q_2)e^P, \end{aligned}$$

*then*$\sigma _2(f)=\deg P$*, where and in the following,*$\deg P$*is the degree of P*.

Mao (2009) studied the problem on Brück conjecture when $f^{(k)}$ is replaced by differential polynomial $L(f)=A_kf^{(k)}+\cdots +A_1f'+A_0f$ in Theorem 1.3.

### **Theorem 1.4**

Mao (2009). *Let P*(*z*) *be a polynomial,*$A_k(z)(\not \equiv 0),\ldots ,A_0(z)$*be polynomials, and f be an entire function of order*

$$\begin{aligned} \sigma (f)>1+\max _{0\le j\le k-1}\left\{ \frac{\deg A_j-\deg A_k}{k-j},0\right\} \end{aligned}$$

*and hyper-order*$\sigma _2(f)<\frac{1}{2}$.* If f and L*(*f*) *share P CM, then*

$$\begin{aligned} \frac{L(f)-P(z)}{f(z)-P(z)}=c, \end{aligned}$$

*for some constant*$c\ne 0$*, where, and in the sequel,*$\deg A_j$*denotes the degree of*$A_j(z)$, *k is a positive integer.*

Chang and Zhu (2009) further investigated the problem related to Brück conjecture and proved that Theorem 1.2 remains valid if the value *a* is replaced by a function *a*(*z*).

### **Theorem 1.5**

[ Chang and Zhu (2009), Theorem 1] *. Let f be an entire function of finite order and a*(*z*) *be a function such that*$\sigma (a)<\sigma (f)<+\infty$. * If f and *$f'$*share a*(*z*) *CM, then *$\frac{f'-a}{f-a}=c$*for some non-zero constant c*.

Thus, an interesting subject arises naturally about this problem: *what would happen when*$\sigma (a)<\sigma (f)<+\infty$*is replaced by*$0<\sigma (a)=\sigma (f)<+\infty$*in Theorems 1.2–1.5?*

## Conclusions

Motivated by the above question, the main purpose of this article is to study the growth of solution of differential equation on entire function *f* and its linear differential polynomial

1 $$\begin{aligned} L_1(f)=a_k(z)f^{(k)}+a_{k-1}(z)f^{(k-1)}+\cdots +a_1(z)f'+a_0(z)f, \end{aligned}$$

where *k* is a positive integer, $a_k(z)(\not \equiv 0),a_{k-1}(z),\ldots ,a_1(z)$ and $a_0(z)$ are polynomials, and obtain the following theorems.

### **Theorem 2.1**

*Let f*(*z*) *and*$\alpha (z)$*be two nonconstant entire functions and satisfy*$0<\sigma (\alpha )=\sigma (f)<+\infty$*and *$\tau (f)>\tau (\alpha )$*, and let P*(*z*) *be a polynomial such that*

2 $$\begin{aligned} \sigma (f)>\deg P+\max \left\{ \frac{deg a_j-deg a_k}{k-j},0\right\} . \end{aligned}$$

*If f is a nonconstant entire solution of the following differential equation*

3 $$\begin{aligned} L_1(f)-\alpha (z)=(f(z)-\alpha (z))e^{P(z)}, \end{aligned}$$

*where*$L_1(f)$*is stated as in* (1). *Then P*(*z*) *is a constant.*

*If *$L_1(f)$*is replaced by the following linear differential polynomial *$L_2(f)$

4 $$\begin{aligned} L_2(f)=a_k(z)f^{(k)}+a_{k-1}(z)f^{(k-1)}+\cdots +a_1(z)f'+a_0(z)f+\beta (z), \end{aligned}$$

where *k* is a positive integer, $a_k(z)(\not \equiv 0),a_{k-1}(z),\ldots ,a_1(z)$ and $a_0(z)$ are polynomials, and $\beta$ is an entire function satisfying either $\sigma (\beta )<\mu (f)$ or $0<\sigma (\beta )=\sigma (f)<+\infty$ and $\tau (\beta )<\tau (f)$, then we obtain the following results.

### **Theorem 2.2**

*Let f*(*z*) *and*$\alpha (z)$*be two nonconstant entire functions and satisfy*$0<\sigma (\alpha )=\sigma (f)<+\infty$*and*$\tau (f)>\tau (\alpha )$*, and let P*(*z) be a polynomial satisfying* (2).* If f is a nonconstant entire solution of the following differential equation*

5 $$\begin{aligned} L_2(f)-\alpha (z)=(f(z)-\alpha (z))e^{P(z)}, \end{aligned}$$

*where*$L_2(f)$*is stated as in* (4)* and*$\beta$*is an entire function satisfying*$0<\sigma (\beta )=\sigma (f)<+\infty$*and*$\tau (\beta )<\tau (f)$. *ThenP*(*z*) *is a constant.*

### **Theorem 2.3**

*Let f*(*z*) *and *$\alpha (z)$*be two nonconstant entire functions and satisfy *$\sigma (\alpha )<\mu (f)$*, and let P*(*z*) *be a polynomial satisfying* (2). *If f is a nonconstant entire solution of Eq. * (5), *where *$L_2(f)$*is stated as in* (4) *and*$\beta$*is an entire function satisfying*$\sigma (\beta )<\mu (f)$*. Then *$\sigma _2(f)=\deg P$.

### **Corollary 2.1**

*Let f*(*z*) *and*$\alpha (z)$*be two nonconstant entire functions and satisfy*$\sigma (\alpha )<\mu (f)$*, and let P*(*z be a polynomial satisfying*) (2). *If f is a nonconstant entire solution of Eq.* (3),*where *$L_1(f)$ is stated as in (1). *Then*$\sigma _2(f)=\deg P$.

## Some Lemmas

To prove our theorems, we will require some lemmas as follows.

### **Lemma 3.1**

Laine (1993). *Let f*(*z*) *be a transcendental entire function,*$\nu (r,f)$*be the central index of f*(*z*). *Then there exists a set*$E\subset (1,+\infty )$*with finite logarithmic measure, we choose z satisfying *$|z|=r\not \in [0,1]\cup E$*and*$|f(z)|=M(r,f)$*, we get*

$$\begin{aligned} \frac{f^{(j)}(z)}{f(z)}=\left\{ \frac{\nu (r,f)}{z}\right\} ^j(1+o(1)), ~~for ~~j\in N. \end{aligned}$$

### **Lemma 3.2**

He and Xiao (1988). *Let f*(*z*)* be an entire function of finite order*$\sigma (f)=\sigma <+\infty$*, and let *$\nu (r,f)$*be the central index of f. Then*

$$\begin{aligned} \limsup _{r\rightarrow +\infty }\frac{\log \nu (r,f)}{\log r}=\sigma (f). \end{aligned}$$

*And if f is a transcendental entire function of hyper order *$\sigma _2(f)$*, then*

$$\begin{aligned} \limsup _{r\rightarrow +\infty }\frac{\log \log \nu (r,f)}{\log r}=\sigma _2(f). \end{aligned}$$

### **Lemma 3.3**

Mao (2009). *Let f be a transcendental entire function and let*$E\subset [1,+\infty )$*be a set having finite logarithmic measure. Then there exists *$\{z_n=r_ne^{i\theta _n}\}$*such that*$|f(z_n)|=M(r_n,f),\theta _n\in [0,2\pi )$, $\lim _{n\rightarrow +\infty }\theta _n=\theta _0\in [0,2\pi ), r_n\not \in E$*and if*$0<\sigma (f)<+\infty$*, then for any given*$\varepsilon >o$*and sufficiently large*$r_n$,

$$\begin{aligned} r_n^{\sigma (f)-\varepsilon }<\nu (r_n,f)<r_n^{\sigma (f)+\varepsilon }. \end{aligned}$$

*If*$\sigma (f)=+\infty$*, then for any given large *$M>0$*and sufficiently large *$r_n$, $\nu (r_n,f)>r_n^M$.

### **Lemma 3.4**

Laine (1993).* Let *$P(z)=b_nz^n+b_{n-1}z^{n-1}+\cdots +b_0$*with *$b_n\ne 0$*be a polynomial. Then, for every *$\varepsilon >0$*, there exists *$r_0>0$*such that for all*$r=|z|>r_0$*the inequalities*

$$\begin{aligned} (1-\varepsilon )|b_n|r^n\le |P(z)|\le (1+\varepsilon )|b_n|r^n \end{aligned}$$

*hold.*

### **Lemma 3.5**

*Let f*(*z*) *and A*(*z*) *be two entire functions with *$0<\sigma (f)=\sigma (A)=\sigma <+\infty , 0<\tau (A)<\tau (f)<+\infty$*, then there exists a set *$E\subset [1,+\infty )$*that has infinite logarithmic measure such that for all *$r\in E$*and a positive number*$\kappa >0$,* we have*

$$\begin{aligned} \frac{M(r,A)}{ M(r,f)}<\exp \{-\kappa r^{\sigma }\}. \end{aligned}$$

### *Proof*

By definition, there exists an increasing sequence $\{r_m\}\rightarrow +\infty$ satisfying $(1+\frac{1}{m})r_m<r_{m+1}$ and

6 $$\begin{aligned} \lim \limits _{m\rightarrow +\infty }\frac{\log M(r_m,f)}{r_m^{\sigma }}=\tau (f). \end{aligned}$$

For any given $\beta (\tau (A)<\beta <\tau (f))$, then there exists some positive integer $m_0$ such that for all $m\ge m_0$ and for any given $\varepsilon (0<\varepsilon <\tau (f)-\beta )$ , we have

7 $$\begin{aligned} \log M(r_m,f)>(\tau (f)-\varepsilon )r_m^{\sigma }. \end{aligned}$$

Thus, there exists some positive integer $m_1$ such that for all $m\ge m_1$, we have

8 $$\begin{aligned} \left( \frac{m}{m+1}\right) ^{\sigma }>\frac{\beta }{\tau (f)-\varepsilon }. \end{aligned}$$

From (6–8), for all $m\ge m_2=\max \{m_0,m_1\}$ and for any $r\in [r_m,(1+\frac{1}{m})r_m]$, we have

9 $$\begin{aligned} M(r,f)&\ge M(r_m,f)>\exp \{(\tau (f)-\varepsilon )r_m^{\sigma }\} \\&\ge \exp \left\{ (\tau (f)-\varepsilon )\left( \frac{m}{m+1}r\right) ^{\sigma }\right\} >\exp \{\beta r^{\sigma }\}.\nonumber \end{aligned}$$

Set $$E=\bigcup _{m=m_2}^{\infty }[r_m,(1+\frac{1}{m})r_m]$$, then

$$\begin{aligned} m_{l}E=\sum _{m=m_2}^{\infty }\int _{r_m}^{(1+\frac{1}{m})r_m}\frac{dt}{t} =\sum _{m=m_2}^{\infty }\log \left( 1+\frac{1}{m}\right) =\infty . \end{aligned}$$

From the definition of type of entire function, for any sufficiently small $\varepsilon >0$, we have

10 $$\begin{aligned} M(r,A)<\exp \{\tau (A)+\varepsilon )r^\sigma \}. \end{aligned}$$

By (9) and (10), set $\kappa =\beta -\tau (A)-\varepsilon$, for all $r\in E$, we have

$$\begin{aligned} \frac{M(r,A)}{M(r,f)}<\exp \{-(\beta -\tau (A)-\varepsilon )r^\sigma \}=e^{-\kappa r^\sigma }. \end{aligned}$$

Thus, this completes the proof of this lemma. $\square$

## The proof of Theorem 2.1

### *Proof*

Since *P*(*z*) is a polynomial, assume that $\deg P=m\ge 1$. Let

$$\begin{aligned} P(z)=b_mz^m+b_{m-1}z^{m-1}+\cdots +b_0, \end{aligned}$$

where $b_m,\ldots ,b_0$ are constants and $b_m\ne 0, m\ge 1$. Thus, it follows from (3) and Lemma 3.4 that

11 $$\begin{aligned} |b_m|r^m(1+o(1))=|P(z)|=\left| \log \frac{\frac{L_1(f(z))}{f(z)}-\frac{\alpha (z)}{f(z)}}{1-\frac{\alpha (z)}{f(z)}}\right| . \end{aligned}$$

Since $L_1(f)=a_kf^k+a_{k-1}f^{(k-1)}+\cdots +a_0f$, from Lemma 3.1, then there exists a subset $E_1\subset (1,+\infty )$ with finite logarithmic measure, such that for some point $|z|=re^{i\theta } (\theta \in [0,2\pi ))$, $r\not \in E_1$ and $M(r,f)=|f(z)|$, we have

$$\begin{aligned} \frac{f^{(j)}(z)}{f(z)}=\left\{ \frac{\nu (r,f)}{z}\right\} ^j(1+o(1)), \quad 1\le j\le k. \end{aligned}$$

Thus, it follows that

12 $$\begin{aligned} \frac{L_1(f(z))}{f(z)}&=a_k\left\{ \frac{\nu (r,f)}{z}\right\} ^k(1+o(1))+\cdots +a_1\left\{ \frac{\nu (r,f)}{z}\right\} (1+o(1))+a_0\nonumber \\&=\frac{a_k}{z^k}(1+o(1))\left[ \nu (r,f)^k+\sum _{j=1}^k\frac{a_{k-j}}{a_k}z^j\nu (r,f)^{k-j}(1+o(1))\right] . \end{aligned}$$

From Lemma 3.3, there exists $\{z_n=r_ne^{i\theta _n}\}$ such that $|f(z_n)|=M(r_n,f),\theta _n\in [0,2\pi )$, $\lim _{n\rightarrow \infty }\theta _n=\theta _0\in [0,2\pi ), r_n\not \in E_1$, then for any given $\varepsilon$ satisfying

$$\begin{aligned} 0<\varepsilon <\min _{1\le j\le k}\frac{j[\sigma (f)-\deg P-\frac{d_{k-j}}{j}]}{3k-j}, \end{aligned}$$

where $d_{k-j}=deg a_{k-j}-deg a_k$, and sufficiently large $r_n$, we have

13 $$\begin{aligned} r_n^{\sigma (f)-\varepsilon }<\nu (r_n,f)<r_n^{\sigma (f)+\varepsilon }. \end{aligned}$$

Since $a_0(z), \ldots , a_k(z)$ are polynomials, let $a_j(z)=\sum _{t=0}^{s_j}l_{jt}z^t$, where $s_j=\deg a_j, j=0,1,\ldots ,k$. Then, from Lemma 3.4 and (13), we have

14 $$\begin{aligned} \left| \frac{a_{k-j}}{a_k}z^j\nu (r,f)^{k-j}(1+o(1))\right|&\le M\frac{|l_{k-j,s_{k-j}}|r_n^{s_{k-j}}}{|l_{k,s_{k}}|r_n^{s_{k}}}r_n^jr_n^{(\sigma (f)+\varepsilon )(k-j)}\nonumber \\&=M\frac{|l_{k-j,s_{k-j}}|}{|l_{k,s_{k}}|}r_n^{d_{k-j}+j+(\sigma (f)+\varepsilon )(k-j)}\nonumber \\&\le M\frac{|l_{k-j,s_{k-j}}|}{|l_{k,s_{k}}|}r_n^{k\sigma (f)-j\sigma (f)+d_{k-j}+\deg P+(k-j)\varepsilon }, \end{aligned}$$

where $d_{k-j}=s_{k-j}-s_k$ and *M* is a positive constant. Since $-j\sigma (f)+d_{k-j}+\deg P+(k-j)\varepsilon <-2k\varepsilon <0$, it follows from (14) that

15 $$\begin{aligned} \left| \frac{a_{k-j}}{a_k}z^j\nu (r_n,f)^{k-j}(1+o(1))\right|&< M\frac{|l_{k-j,s_{k-j}}|}{|l_{k,s_{k}}|}r_n^{k(\sigma (f)-2\varepsilon )}\nonumber \\&= o( \nu (r_n,f)^{k}), ~~as~~r_n\rightarrow +\infty , ~~r_n\not \in E_1. \end{aligned}$$

Since $0<\sigma (\alpha )=\sigma (f)<+\infty$ and $\tau (\alpha )<\tau (f)<+\infty$, from Lemma 3.5, there exists a set $E\subset [1,+\infty )$ that has infinite logarithmic measure such that for a sequence $\{r_n\}_1^\infty \in E_2=E-E_1$, we have

16 $$\begin{aligned} \frac{M(r,\alpha )}{ M(r,f)}<\exp \{-\kappa r_n^{\sigma (f)}\}\rightarrow 0, \quad as~~n\rightarrow +\infty . \end{aligned}$$

From (11), (12), (15), (16) and Lemma 3.2, we can get that

17 $$\begin{aligned} |b_m|r_n^m(1+o(1))=|P(z)|=O(\log r_n), \end{aligned}$$

which is impossible. Thus, *P*(*z*) is not a polynomial, that is, *P*(*z*) is a constant.

Thus, this completes the proof of Theorem 2.1. $\square$

## The proof of Theorem 2.2

### *Proof*

First of all, we rewrite (5) as

18 $$\begin{aligned} \frac{L_2(f)-\alpha (z)}{f(z)-\alpha (z)} =\frac{\frac{L_1(f)}{f}+\frac{\beta (z)}{f(z)}-\frac{\alpha (z)}{f(z)}}{1-\frac{\alpha (z)}{f(z)}}=e^{P(z)}, \end{aligned}$$

where $L_1(f)$ is stated as in Theorem 2.1. Since $0<\sigma (f)=\sigma (\alpha )=\sigma (\beta )<+\infty$, $\tau (\alpha )<\tau (f)$ and $\tau (\beta )<\tau (f)$, from Lemma 3.5, there exists a set $E\subset [1,+\infty )$ that has infinite logarithmic measure such that for a sequence $\{r_n\}_1^\infty \in E_3=E-E_1$, we have

$$\begin{aligned} \frac{M(r,\alpha )}{M(r,f)}<\exp \{-\kappa r_n^{\sigma (f)}\}\rightarrow 0, ~~and ~~\frac{M(r,\beta )}{M(r,f)}<\exp \{-\kappa r_n^{\sigma (f)}\}\rightarrow 0,~~~as ~~n\rightarrow +\infty . \end{aligned}$$

Then by using the proceeding as in proof of Theorem 2.1, we prove that *P*(*z*) is a constant.

This completes the proof of Theorem 2.2. $\square$

## The proof of Theorem 2.3.

### *Proof*

From *P*(*z*) is a polynomial, we will consider two cases (i) $\sigma (f)<+\infty$ and (ii) $\sigma (f)=+\infty$.

*Case 1.* Suppose that $\sigma (f)<+\infty$. Then $\sigma _2(f)=0$. Since $\sigma (\alpha )<\mu (f), \sigma (\beta )<\mu (f)$, from Definitions of the order and the lower order, there exists infinite sequence $\{z_n\}_1^\infty$, we have

$$\begin{aligned} \frac{|\alpha (z_n)|}{|f(z_n)|}\rightarrow 0, ~~and ~~\frac{|\beta (z_n)|}{|f(z_n)|}\rightarrow 0,~~~as ~~n\rightarrow \infty . \end{aligned}$$

Thus, by using the same argument as in Theorem 2.1, we can get that *P*(*z*) is a constant, that is, $\deg P=0$. Therefore, $\sigma _2(f)=\deg P$.

*Case 2.* Suppose that $\sigma (f)=+\infty$. Set $F(z)=f(z)-\alpha (z)$. Since $\sigma (\alpha )<\mu (f)$, it follows from (2) that

19 $$\begin{aligned} \sigma (F)=+\infty , ~~\sigma _2(F)=\sigma _2(f), \end{aligned}$$

and

20 $$\begin{aligned} \sigma (F)>\deg P+\max \left\{ \frac{deg a_j-deg a_k}{k-j},0\right\} . \end{aligned}$$

Furthermore, we can rewrite (4) as

21 $$\begin{aligned} a_k(z)\frac{F^{(k)}(z)}{F(z)}+\cdots +a_1(z)\frac{F'(z)}{F(z)}+a_0(z)+\frac{\gamma (z)}{F(z)}=e^{P(z)}, \end{aligned}$$

where $\gamma (z)=a_k\alpha ^{(k)}+\cdots +a_1\alpha +\beta -\alpha$. Since $\sigma (\beta )<\mu (f)$, $\sigma (\alpha )<\mu (f)$ and $a_i(z), (i=0,\ldots ,k)$ are polynomials, we have

22 $$\begin{aligned} \sigma (\gamma )\le \max \{\sigma (\alpha ),\sigma (\beta )\}<\mu (f)\le \sigma (f). \end{aligned}$$

From Lemma 3.1, there exists a set $E_4\subset (1,+\infty )$ with finite logarithmic measure, we choose *z* satisfying $|z|=r\not \in [0,1]\cup E_4$ and $|F(z)|=M(r,F)$, we get

23 $$\begin{aligned} \frac{F^{(j)}(z)}{F(z)}=\left\{ \frac{\nu (r,F)}{z}\right\} ^j(1+o(1)), ~~for ~~j\in 1,2,\ldots ,k. \end{aligned}$$

Since $\sigma (F)=+\infty$, then it follows from Lemma 3.3 that there exists $\{z_n=r_ne^{i\theta _n}\}$ with $|F(z_n)|=M(r_n,F),\theta _n\in [0,2\pi )$, $\lim _{n\rightarrow \infty }\theta _n=\theta _0\in [0,2\pi ), r_n\not \in E_5$, such that for any large constant *K* and for sufficiently large $r_n$ we have

24 $$\begin{aligned} \nu (r_n,F)\ge r_n^K. \end{aligned}$$

From $M(r_n,F)=|F(z_n)|$, $F(z), \gamma (z)$ are entire functions and (18), by using definitions of the order and the lower order, we have

25 $$\begin{aligned} \left| \frac{\gamma (z_n)}{F(z_n)}\right| \rightarrow 0, ~~~as ~~r\rightarrow +\infty . \end{aligned}$$

Thus, it follows from (21), (23)–(25) that

26 $$\begin{aligned} a_k\left( \frac{\nu (r_n,F)}{z_n}\right) ^k(1+o(1))=e^{P(z_n)}. \end{aligned}$$

Let

$$\begin{aligned} P(z)=b_mz^m+b_{m-1}z^{m-1}+\cdots +b_0, \end{aligned}$$

where $b_m,\ldots ,b_0$ are constants and $b_m\ne 0, m\ge 1$. From Lemma 3.4, there exists sufficiently large positive number $r_0$ and $n_0\in \mathbb {N}_+$, such that for sufficiently large positive integer $n>n_0$ satisfying $|z_n|=r_n>r_0$, we have for every $\varepsilon '>0$

27 $$\begin{aligned} \log |b_m|+m\log |z_n|+\log |1-\varepsilon '|\le \log |P(z_n)|\le |\log \log e^{P(z_n)}|. \end{aligned}$$

It follows from (26) that

28 $$\begin{aligned} |\log \log e^{P(z_n)}|&\le \log |\log |a_k||+\log \log \nu (r_n,F)+\log \log r_n+O(1)\nonumber \\&\le \log \log \nu (r_n,F)+O(\log \log r_n). \end{aligned}$$

Thus, we have from (27), (28) and Lemma 3.2 that

29 $$\begin{aligned} m=\deg P(z)\le \sigma _2(F)=\sigma _2(f). \end{aligned}$$

On the other hand, since $a_k$ is a polynomial, it follows from (27) and Lemma 3.4 that

$$\begin{aligned} M(r_n,e^{P(z_n)})\ge K_1r_n^{d_k}\left( \frac{\nu (r_n,F)}{r_n}\right) ^k, \end{aligned}$$

where $K_1>0$ is a constant. Then we have

30 $$\begin{aligned} \nu (r_n,F)^k\le K_1^{-1}r_n^{k-d_k}M(r_n,e^{P(z_n)}). \end{aligned}$$

Thus, it follows from (30) and Lemma 3.2 that

$$\begin{aligned} \sigma _2(f)&=\sigma _2(F)=\limsup _{r_n\rightarrow +\infty }\frac{\log \log \nu (r_n,F)}{\log r_n} =\limsup _{r_n\rightarrow +\infty }\frac{\log \log \nu (r_n,F)^k}{\log r_n}\\&\le \limsup _{r_n\rightarrow +\infty }\frac{\log \log K_1^{-1}r_n^{k-d_k}M(r_n,e^{P(z_n)})}{\log r_n}=\sigma (e^{P}). \end{aligned}$$

Since *P*(*z*) is a polynomial, then $\sigma (e^{P})=\deg P=m$. By combining (29), we have $\sigma _2(f)=\deg P$.

Therefore, this completes the proof of Theorem 2.3. $\square$
